# Hyperthermic treatment of DMBA-induced rat mammary cancer using magnetic nanoparticles

**DOI:** 10.1186/1477-044X-6-2

**Published:** 2008-02-25

**Authors:** Jun Motoyama, Noriyuki Yamashita, Tomio Morino, Masashi Tanaka, Takeshi Kobayashi, Hiroyuki Honda

**Affiliations:** 1 Nanotherapy Co., Ltd, 19-11, Kikui 2-chome, Nishi-ku, Nagoya 451-0044, Japan; 2 Department of Biotechnology, School of Engineering, Nagoya University, Furo-cho, Chikusa-ku, Nagoya 464-8603, Japan; 3 Department of Biological Chemistry, School of Bioscience and Biotechnology, Chubu University, 1200 Matsumoto-cho, Kasugai, Aichi 487-8501, Japan

## Abstract

**Background:**

We have developed magnetite cationic liposomes (MCLs) and applied them as a mediator of local hyperthermia. MCLs can generate heat under an alternating magnetic field (AMF). In this study, the in vivo effect of hyperthermia mediated by MCLs was examined using 7,12-dimethylbenz(a)anthracene (DMBA)-induced rat mammary cancer as a spontaneous cancer model.

**Method:**

MCLs were injected into the mammary cancer and then subjected to an AMF.

**Results:**

Four rats in 20 developed mammary tumors at more than 1 site in the body. The first-developed tumor in each of these 4 rats was selected and heated to over 43°C following administration of MCLs by an infusion pump. After a series of 3 hyperthermia treatments, treated tumors in 3 of the 4 rats were well controlled over a 30-day observation period. One of the 4 rats exhibited regrowth after 2 weeks. In this rat, there were 3 sites of tumor regrowth. Two of these regrowths were reduced in volume and regressed completely after 31 days, although the remaining one grew rapidly. These results indicated hyperthermia-induced immunological antitumor activity mediated by the MCLs.

**Conclusion:**

Our results suggest that hyperthermic treatment using MCLs is effective in a spontaneous cancer model.

## Background

Magnetic nanoparticles have been widely used in biological and medical fields. For instance, magnetic separation is used to separate certain biomaterials [1] and/or cells [2,3] by using biologically labeled magnetic beads. In the medical field, certain types of magnetic dispersion are used as a contrast agent in magnetic resonance imaging (MRI) diagnosis [4]. Recently, magnetic nanoparticles have also been investigated for therapeutic purposes such as hyperthermic treatment [5].

Hyperthermic treatments have been used for many years, particularly in anticancer therapy [6]. Typically, there are 2 ranges of targeting temperature used in such treatment. High temperatures (greater than 50°C) kill targeted tissue directly. Such direct heating methods using needle-type interstitial antenna materials are effective for inducing hyperthermia in local regions; however, these technologies are less effective for larger areas of tissue [7]. Lower temperatures of approximately 40°C to 43°C are also associated with an anticancer effect. There are several heating techniques employed in hyperthermic treatments using such a temperature range. For example, radiofrequency (RF) electric field application is one of the approved methods for cancer treatment. However, the effectiveness of this technique varies according to tumor size and the depth of the tumor region from the body surface. Furthermore, it is sometimes difficult to focus on the exact location of the tumor [8]. Microwaves, ultrasound applied as a heating method, are also used for inducing hyperthermia. For such hyperthermic therapy, magnetic nanoparticles have also used been used as a heating mediator. The use of these particles is based on fact that such multidomain ferro- or ferri-magnetic materials are heated by an alternating magnetic field due to hysteresis losses [9]. Single domain particles of magnetite can also generate heat by relaxation loss, not hysteresis loss, under irradiation of alternating magnetic field. Ten nanometer of diameter is boundary size to allocate particles between single-domain and multi-domain. Optimal diameter of magnetic nanoparticle for magnetic nanoparticle-mediated hyperthermia should be investigated in detail.

Our group has pioneered one of these technologies in order to selectively heat tumor regions [10]. We have introduced cationic liposome technology in order to enhance the surface interaction between cells and heating mediator, and thereby improve its localization. In previous animal studies, we have demonstrated the efficacy of hyperthermia induce using magnetite cationic liposomes (MCLs) [10-12] in several types of tumor model; for instance, B16 melanoma in mice [13,14], T9 glioma in rats [12,15], osteosarcoma in hamsters [16], MM46 mouse mammary carcinoma [17], PLS 10 rat prostate cancer [18], and VX-7 squamous cell carcinoma in rabbit tongue [19]. Furthermore, we have described the capability of immunologic reactivity in cancer therapy, which was enhanced by increasing the amount of heat-shock protein 70 (HSP70), following hyperthermic treatment with MCLs [20-22].

In several transplantable tumor models mentioned above, the magnetite nanoparticle-mediated hyperthermia proposed by us was found to be very effective for inducing complete tumor regression. In such models, however, transplantable cell is an explanted cell. To use the hyperthermia for human patients, the effect of the hyperthermia should be demonstrated by the use of more practical tumor model. We focused on oncogene transgenic mice as spontaneous tumor model and complete tumor regression of malignant melanoma induced by the oncogene ret was achieved by the hyperthermia. As a next step, we investigated here the application to carcinogen-induced spontaneous tumor models as a practical tumor model which can simulate practical tumor in the human patient.

7,12-Dimethylbenz(a)anthracene (DMBA)-induced rat mammary cancer has been widely exploited in cancer studies for many years, since it was first reported by Huggins et al. [23]. This model is very useful as a spontaneous cancer model, since blood vessels and lymphoducts surrounding the tumor tissue are likely to simulate human cancer. In this study, we applied our hyperthermic treatment system with the MCLs to DMBA-induced rat mammary cancer model.

## Materials and methods

### Animals and mammary tumor models

Eight-week-old Sprague-Dawley (SD) rats were purchased from the Charles River Laboratory Japan, Inc. (Kanagawa, Japan). DMBA, purchased from Wako Pure Chemical Industry, Ltd. (Osaka, Japan), was dissolved in sesame oil, purchased from ICN Biomedicals, Inc.(CA, USA), and the concentration was adjusted to 40 mg/mL.

A single dose of 0.5 mL (20 mg DMBA/body) of DMBA/sesame oil solution was administered to 20 rats via esophageal intubation. After 10 weeks, 4 rats developed mammary tumors at more than 1 site in the body. In the each of these rats, we selected 1 tumor region that exceeded 10 mm in its long axis and used this for the experiments.

All animal experiments were conducted in accordance with the "Guide for the care and use of laboratory animals of Nagoya University."

### Preparation of the MCLs

Magnetite nanoparticles (average diameter, 10 nm) were purchased from Toda Kogyo Co. (Hiroshima, Japan). N-(α-trimethyl-ammonioacetyl) didodecyl-D-glutamate chloride (TMAG) was purchased from Sogo Pharmaceutical Co. (Tokyo, Japan). Dilauroyl-phosphatidylcholine (DLPC) and dioleoylphos-phatidylethanolamine (DOPE) were purchased from NOF Co. (Tokyo, Japan).

The MCLs were prepared by using a previously described sonication method, with slight modification [10]. Briefly, 1 mL of colloidal magnetite nanoparticle dispersion was mixed with lipid membrane that consisted of the abovementioned phospholipids. Here, the molar ratio of TMAG, DLPC, and DOPE was 1:2:2. The magnetite concentration in the MCLs was determined by measuring iron content using the potassium thiocyanate method [24]. The net concentration of magnetite in the MCLs was 20 mg/mL.

### Hyperthermia treatment

All 4 rats developed mammary tumors discretely at more than 1 site in the body. In each rat, we selected the first-developed tumor for the hyperthermic treatment. The MCLs were infused gradually into the tumor directly in approximately 30 minutes by using an infusion pump (SP100i; World Precision Instruments Inc., FL, USA). Depending on the tumor volume, we determined the injection volume and the number of injection sites required to obtain a uniform distribution of the MCLs. The applied dose of the MCLs was approximately 2 mg MCL per mL tumor volume. After the injection, the rats were subjected to the first hyperthermia treatment for 30 minutes under anesthesia. This treatment was repeated on the following 2 days. In order to raise the temperature of the tumor region, the rats were bedded above an irradiation coil. An alternating magnetic field (AMF) was applied using an AMF radiator (HI-HEATER 5010; DHF Co. Ltd., Tokyo, Japan). The magnetic field frequency used was 360 kHz. The temperatures at the surface of the tumor tissue and rectum were measured by optical fiber probe thermometers (FX-9020; Anritsu Meter Co., Tokyo, Japan).

We started with the power of the AMF irradiator at 1.6 kW, therefore, SAR value of the MCL was about 175 W/g-magnetite. Thereafter, we controlled the temperature of tumor site by manipulating volume of the irradiating power. We observed quick response of the heat value by turning of the irradiating.

## Results

### Tumor temperature induced by the hyperthermia treatment

**Figure 1:**
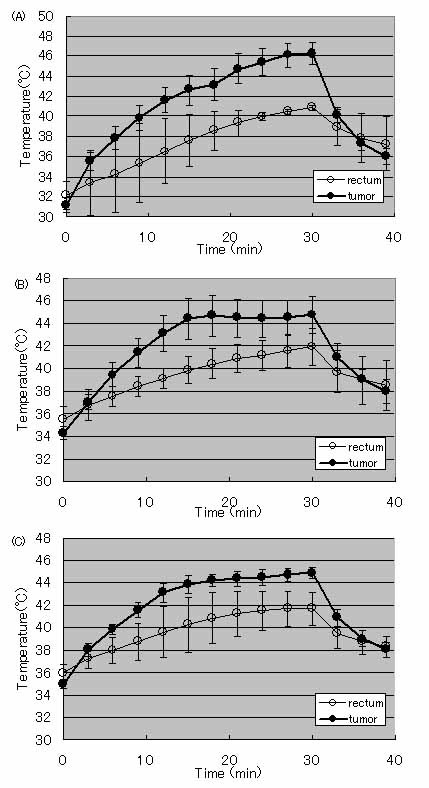
**Temperature profiles of the surface of the tumor (closed circles) and rectum (open circles) during the hyperthermia treatment at day 1 (A), 2 (B), and 3 (C).** Data points and bars represent the mean and SD of 4 treated rats.

Figure 1 shows the mean temperature profiles at the site of the tumor surface and rectum during the hyperthermia treatment on each treatment day. The temperature of the tumor region rose depending on the applied AMF, reached 45°C, and was then maintained at ± 2°C for the remainder of the treatment. The rectum temperature also increased gradually but it was lower than that of tumor surface and was not dependent on the strength of the AMF field. The temperature difference between two regions was 2–3°C. After termination of the irradiation treatment, both temperatures decreased immediately.

### Tumor volume monitoring

**Figure 2:**
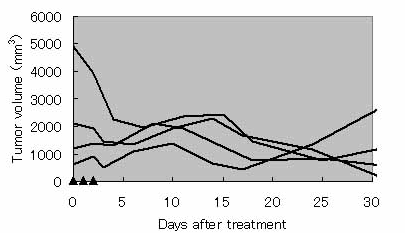
Tumor growth profiles at the treatment sites after 3 treatments. Triangles represent treatment days.

**Figure 3:**
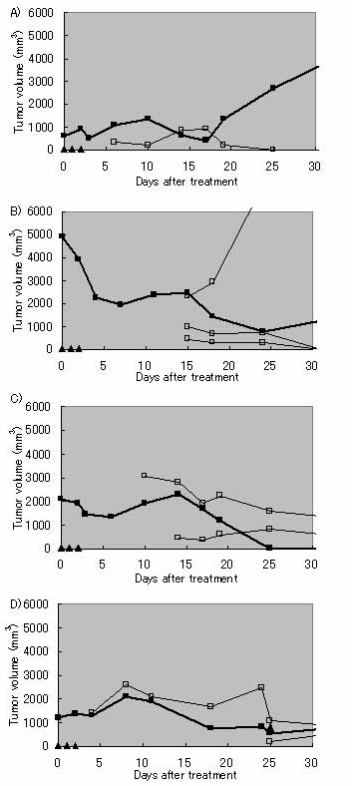
**Tumor growth profiles for each rat after 3 treatments (A, Rat #1; B, #2; C, #3; and D, #4).** Closed squares: treated tumor; open squares: untreated tumor. Triangles represent treatment days.

**Figure 4:**
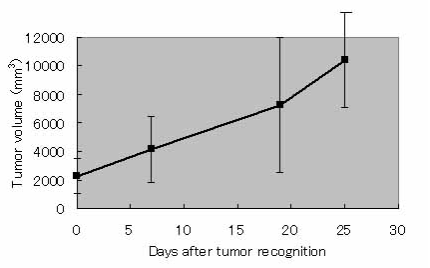
**Tumor growth profiles without any kind of treatment after it came out to be recognizable.** The data was gathered by additional study, and was averaged of 4 individual rats

Figure 2 shows the tumor volume profiles of treated tumors after a series of 3 hyperthermia treatments. In 3 of the 4 rats, the sizes of the treated tumors were well controlled over a 30-day observation period; the remaining tumor was controlled for only 3 weeks and regrew thereafter. Figure 3 shows tumor volume profiles after the treatment in each rat. In all 4 rats, we observed multiple growth of tumors at 1–3 sites in the body. Although those tumor areas were untreated, their growth volumes were also measured. In rat #2, there were 3 sites of untreated sites of the tumor, 1 site of which grew rapidly. The others were, however, reduced in volume and had regressed completely after 31 days. In other rats, untreated sites of tumor were also reduced in volume, or well controlled. In Figure 4, tumor growth of untreated group of rats were shown after it sizes comes out to be recognizable. Without any treatment, DMBA-induced tumors grew rapidly on the average basis of 4 individual rats.

## Discussion

Human breast cancer usually originates in the ductal region and the DMBA-induced mammary tumor model exhibits the same origin [25]. Therefore, this rat model is considered to be a good animal model of human breast cancer.

Figure 1 shows that the temperature increases in the tumor region were slightly slower than those previously reported by our group [16]. We believe that this difference is due to the cooling effect of the vascular systems that developed in the spontaneous tumor tissue. However, it was possible to raise the temperature of the tumor region during the treatment periods, and we therefore followed former protocols in this study.

After the course of treatment, all 4 rats that developed multiple mammary tumors exhibited tumor regression in the immediate region of the treated site. In spite of this being our first attempt to apply our protocol to a hyperthermia effect similar to that obtained for a transplanted tumor model that we reported previously.

As shown in Fig. 2, 1 of the 4 rats exhibited rapid regrowth the tumor within 2 weeks. Another rat exhibited slight regrowth. These results appear to indicate certain inadequacies of the present treatment protocol as applied to the spontaneous tumor model, despite it being applicable for the previously investigated transplanted tumor model. One of the possible reasons for this discrepancy is considered to be the highly developed vasculature and/or lymphoid system in the spontaneous tumors that were not present in transplanted tumors. A highly developed vasculature might create localized regions of tissue that do not reach sufficiently high temperatures. The developed nutrition/elimination systems also appear to promote the clearance of the administered MCLs from the injected region. Therefore, it is suggested that the treatment protocol should be optimized, particularly with respect to the dose of MCL, the temperature during treatment, and the number of repeat treatments. Given such optimization, it is expected that such regrowths would be repressed. However, tumor growth was remarkably controlled in the early going compared with untreated group of rats (Fig. 4). Therefore, we believe our hyperthermia system will be capable in the future.

In the case of the carcinogen-induced spontaneous tumor models, it is considered that the sites of origin in the animal body are heterogeneous. Indeed, in our experiments, tumor growth at several untreated sites progressed regardless of the treatment. Meanwhile, we also observed some tumor shrinkage at sites where no MCLs had been administered, and which had therefore not been exposed to the hyperthermic conditions during the treatment. These shrinkages were maintained stably for more than 50 days without additional treatment. These results suggest the presence of an immunological antitumor reaction induced by the MCL-mediated hyperthermia, as observed previously with a transplanted tumor model [20]. These responses are believed to be linked to a specific antigen, such as HSP70, that is generated by necrosis of the directly heated tumor cells [21,22]. We also reported the mechanisms of immune reactions which are induced by our hypertharmia system in the previous study [26,27]. We demonstrated the mechanism of immunological reaction which is enhanced by HSP70 dumped from dead tissue with our hyperthermia system. It was also revealed that cancer specific DC maturation was induced by our treatment system.

In the present study, we therefore confirmed the advantage of our hyperthermic procedure in the treatment of carcinogen-induced spontaneous tumors. In the future, it will be necessary to optimize operational conditions in order to enhance the hyperthermic effect in the spontaneous tumor model.

## Authors' contributions

JM carried out the preparation of MCL and animal experiment, NY carried out animal experiment, TM conceived of the study and participated in its design, MT participated as an expert of animal experiment, TK participated in its design and coordination, HH participated in its design and drafted the manuscript together with JM. All authors read and approved the final manuscript.

## References

[B1] Safarik I, Safarikova M (2004). Magnetic techniques for the isolation and purification of proteins and peptides. Biomagn Res Technol.

[B2] Miltenyi S, Mullar W, Weichel W, Radbruch A (1990). High gradient magnetic cell separation with MACS. Cytometry.

[B3] Radbruch A, Mechtold B, Thiel A, Miltenyi S, Pfluger E (1994). High-gradient magnetic cell sorting. Methods Cell Biol.

[B4] Dooms GH, Hicak H, Crooks LE, Higgins CB (1984). Magnetic resonance imaging of the lymph nodes. Radiology.

[B5] Häfeli U, Schütt W, Teller J, Zborowski M (1997). Scientific and Clinical Application of Magnetic Carriers.

[B6] Overgaard J (1985). Hyperthermic Oncology.

[B7] Kobayashi T, Kida Y, Tanaka T, Hattori K, Matsui M, Amemiya Y (1984). Interstitial hyperthermia of malignant brain tumors by implant heating system: Clinical experiment. J Neurooncol.

[B8] Kato H, Ishida T (1993). Present and future status of noninvasive selective deep heating using RF in hyperthermia. Medical & Biological Engineering & Computing, Kyoto World Congress Supplement.

[B9] Andrä W, Nowak H (1998). Magnetism in Medicine: A Handbook, Wiley-VCH, New York.

[B10] Shinkai M, Yanase M, Honda H, Wakabayashi T, Yoshida J, Kobayashi T (1996). Intracellular hyperthermia for cancer using magnetite cationic liposomes: in vitro study. Jpn J Cancer Res.

[B11] Yanase M, Shinkai M, Honda H, Wakabayashi T, Yoshida J, Kobayashi T (1997). Intracellular hyperthermia for cancer using magnetite cationic liposomes: ex vivo study. Jpn J Cancer Res.

[B12] Yanase M, Shinkai M, Honda H, Wakabayashi T, Yoshida J, Kobayashi T (1998). Intracellular hyperthermia for cancer using magnetite cationic liposomes: an in vivo study. Jpn J Cancer Res.

[B13] Suzuki M, Shinkai M, Honda H, Kobayashi T (2003). Anti-cancer effect and immune induction by hyperthermia of malignant melanoma using magnetite cationic liposomes. Melanoma Res.

[B14] Shinkai M, Yanase M, Suzuki M, Honda H, Wakabyashi T, Yoshida J, Kobayashi T (1999). Intracellular hyperthermia for cancer using magnetic cationic liposomes. J Magn, MagMater.

[B15] Ito A, Tanaka K, Shinkai M, Honda H, Matumoto K, Saida T, Kobayashi T (2003). Tumor regression by combined immunotherapy and hyperthermia using magnetic nanoparticles in an experimental subcutaneous murine melanoma. Cancer Sci.

[B16] Matsuoka F, Shinkai M, Honda H, Kubo T, Sugita T, Kobyashi T (2004). Hyperthermia using magnetite cationic liposomes for hamster osteosarcoma. BioMag Res Tech.

[B17] Ito A, Tanaka K, Honda H, Abe S, Yamaguchi H, Kobayashi T (2003). Complete regression of mouse mammary carcinoma with a size greater than 15 mm by frequent repeated hyperthermia using magnetite nanoparticles. J Biosci Bioeng.

[B18] Kawai N, Ito A, Nakahara Y, Futakuchi M, Shirai T, Honda H, Kobayashi T, Kohri K (2005). Anticancer effect of hyperthermia on prostate cancer mediated by magnetite cationic induction in transplanted syngenic rats. Prostate.

[B19] Matsuno H, Tohnai I, Mitsudo K, Hayashi Y, Ito M, Shinkai M, Kobayashi T, Yoshida J, Ueda M (2001). Interstitial hyperthermia using magnetite cationic liposomes inhibit to tumor growth of VX-7 transplanted tumor in rabbit tongue. Jpn J Hyperthermic Oncol.

[B20] Yanase M, Shinkai M, Honda H, Wakabayashi T, Yoshida J, Kobayashi T (1998). Antitumor immunity induction by intracellular hyperthermia using magnetite cationic liposomes. Jpn J Cancer Res.

[B21] Ito A, Shinkai M, Honda H, Wakabayashi T, Yoshida J, Kobayashi T (2001). Augmentation of MHC class I antigen presentation via heat shock protein expression by hyperthermia. Cancer Immunol Immunother.

[B22] Ito A, Shinkai M, Honda H, Yoshikawa K, Saga S, Wakabayashi T, Yoshida J, Kobayashi T (2003). Heat shock protein 70 expression induces antitumor immunity during intracellular hyperthermia using magnetite nanoparticles. Cancer Immunol Immunother.

[B23] Huggins C, Grand LC, Brillantes FP (1961). Mammary cancer induced by a single feeding of polynuclear hydrocarbons and its suppression. Nature.

[B24] Shinkai M, Le B, Honda H, Yoshikawa K, Shimizu K, Saga S, Wakabayashi T, Yoshida J, Kobayashi T (2001). Targeting hyperthermia for renal cell carcinoma using human MN antigen-specific magnetoliposomes. Jpn J Cancer Res.

[B25] Welsch CW (1985). Host factors affecting the growth of carcinogen-induced rat mammary carcinomas: A review and tribute to Charles Brenton Huggins. Cancer Res.

[B26] Tanaka K, Ito A, Kobayashi T, Kawamura T, Shimada S, Matsumoto K, Saida T, Honda H (2005). Intratumoral injection of immature dendritic cells enhances antitumor effect of hyperthermia using mgnetic nanoparticles. Int J Cancer.

[B27] Ito A, Honda H, Kobayashi T (2007). Cancer immnotherapy based on intracellular hyperthermia using magnetite nanoparticles: a novel concept of "heat-controlled necrosis" with heat shock protein expression. Cancer Immunol Immunother.

